# Implementing psychiatric day treatment for infants, toddlers, preschoolers and their families: a study from a clinical and organizational perspective

**DOI:** 10.1186/1752-4458-7-12

**Published:** 2013-04-20

**Authors:** Tilman Furniss, Jörg M Müller, Sandra Achtergarde, Ida Wessing, Marlies Averbeck-Holocher, Christian Postert

**Affiliations:** 1 Department of Child and Adolescent Psychiatry, University Hospital Münster, Schmeddingstr. 50, Münster, 48149, Germany

**Keywords:** Infant, Toddler and preschooler mental health, Preschool Family Day Hospital, Parent–child interaction therapy, Adults as psychiatric patients in child psychiatry

## Abstract

**Background:**

An increasing number of empirical studies indicate that infants, toddlers and preschoolers may suffer from non-transient mental illnesses featuring developmental psychopathology. A few innovative child psychiatric approaches have been developed to treat infants, toddlers and preschoolers and their families, but have not yet been conceptually presented and discussed in the framework of different healthcare systems. The organizational and clinical experience gained while developing specific approaches may be important across disciplines and guide future developments in psychiatric treatment of infants, toddlers, preschoolers and their families.

**Results:**

This article introduces the Preschool Family Day Hospital for Infants, Toddlers and Preschoolers and their Families at Münster University Hospital, Germany. This hospital is unique in the German healthcare system with regard to its social-service institution division of labor. Specifically, it uses an intermittent treatment approach and an integrated interactional family psychiatric approach to treat children and their parents as separate patients. This multidisciplinary, developmentally and family-oriented approach includes components of group treatments with children and separate treatments with parents. Specific techniques include video-assisted treatments of the parent–child interaction, psychiatric and psychotherapeutic treatments for parents, and conjoint family therapies that include both parents and siblings.

**Conclusions:**

The Family Day Hospital for infants, toddlers and preschoolers and their families offers innovative family-oriented treatments for those who suffer from a wide range of severe child psychiatric disorders that cannot be sufficiently treated in outpatient settings. Treatment is based on the need for family-oriented approaches to the early psychiatric treatment of infants, toddlers and preschoolers. Family day hospitals are an innovative approach to preschool child psychiatry that requires further evaluation.

## Background

An increasing number of empirical studies indicate that infants, toddlers and preschoolers may suffer from non-transient mental illnesses featuring developmental psychopathology [1,2] In addition, recent epidemiologic studies have shown that severe internalizing and externalizing mental health symptoms in the clinical range are present in 12%-18% of all preschoolers. This rate is comparable to the prevalence in school-age children [2-5].

Unfortunately, child psychiatric disorders in young children are often underdiagnosed due to insufficient knowledge regarding the symptoms of early mental health disorders and the lack of adequate diagnostic categories and instruments [6]. Children with mental illnesses need age-appropriate psychiatric assessments and treatments; furthermore, these interventions should begin as early as possible to maximize treatment effects and minimize negative, long-term consequences [7,8]. This need for treatment is particularly important in light of the neurobiological research on brain development in the first years of life that suggests that the plasticity of the developing brain is vulnerable to not only deleterious and damaging psychopathological developments but also open to protective and healing influences [9,10].

Research on mother-child relationships and attachment [11-13], parental mental health [14], and neurobiology [15-17] identifies the pivotal role that parents play in the development of mental illness in infants, toddlers and preschoolers. Given this important role, some child psychiatric units have developed a family psychiatry approach to include the parents in the treatment of their psychiatrically ill child [18,19]. This treatment approach presumes that the psychopathology of one family member affects the mental health of others; thus, family members must be included as important contextual factors in the treatment of an index patient. One underlying assumption is that a lack of parental sensitivity, emotional availability or executive functioning leads to affective and behavioral dysregulation and symptom formation in young children [20-22]. Alternatively, developmental deviations and mental health symptoms can create difficulties in attachment and parent–child interactions [23]. Day hospitals for infants, toddlers and preschoolers offer family-oriented treatments for those who suffer from a wide range of severe child psychiatric disorders that cannot be sufficiently treated in outpatient settings [24]. These hospitals exist between the traditional extremes of outpatient and inpatient treatments [8] and attempt to combine the advantages of both settings. Both parents and children participate in intensive multi-modal therapies in a clinical setting; however, children are not separated from their families. The evaluation of treatment outcomes in child psychiatric family day hospitals has yielded promising results with regard to symptom improvements in numerous patients [25,26]. These results support the notion that family day hospitals are an effective approach to child psychiatry; however, this claim requires additional clinical development and evaluation.

Different treatment approaches have been developed for various international healthcare systems. The knowledge gained from specific clinical approaches might be important across disciplines, but it is not often available to the general public. Therefore, we present here the Preschool Family Day Hospital treatment approach developed at Münster University Hospital, Germany. Established in 1997, this center was the first psychiatric day hospital in Germany for infants, toddlers, preschoolers and their parents. This hospital is unique in the German healthcare system with regard to its social service institution division of labor, intermittent treatment concept and integrated family interaction psychiatric approach to treating children and their parents with psychiatric disorders in parallel but separately.

The development of the European family day hospital approach began in the 1960s and 1970s with the establishment of the “Triangel” family day unit in Amsterdam, the Netherlands [27]. Subsequently, numerous child psychiatric family day hospitals were created in England [28-33], Switzerland [26], Finland [21] and Norway [24]. The first German psychiatric family day hospital for infants, toddlers and preschoolers (i.e., under six years) was established in 1997 at the Department of Child Psychiatry at the University of Münster Hospital. Importantly, this specific type of child–parent inpatient treatment must be distinguished from the mother-infant units in adult psychiatry that are primarily intended to treat adult psychiatric disorders such as postpartum depression or postpartum psychosis [34-36]. In contrast, children whose persistent mental illness is the only parameter for admission are always the primary index patients at child psychiatric family day hospitals. Since 1997, several hospitals in Germany implemented treatment facilities for preschool children and their families on a day clinic or inpatient basis.

### Case description: Preschool Family Day Hospital

#### Preschool Family Day Hospital in the German healthcare system

The Preschool Family Day Hospital is part of the Department of Child and Adolescent Psychiatry at the University of Münster Hospital. This unit focuses on the early treatment of children with psychiatric disturbances from infancy to preschool. The unit offers ten treatment places for child psychiatric patients and their parents. One or both of the parents are always involved in their child’s treatment either as accompanying persons or, in case of a concurrent mental illness in the parent, as individually referred psychiatric patients in their own right. The department also offers adult psychiatric expertise and closely cooperates with the adult psychiatric department and the department of psychotherapy at the University of Münster hospital. All German health insurance systems officially recognize parental involvement in their children’s psychiatric treatment [37]. This decree allows parents to be included in their children’s therapy.

Due to the urgent court requirements and statutory responsibilities of social and child protective services, many family day hospitals for preschoolers play decisive roles as assessment units. Different units have been created in Münster to keep these functions institutionally separate. For example, an independent social services family inpatient unit was created to deal primarily with teenage parents and problem families with children at risk who needed parenting, educational and social support assessment. In addition, a separate therapeutic unit to treat victims of child abuse and neglect as well as evaluate possible child sexual abuse was established. Therefore, child psychiatric interventions at the Preschool Family Day Hospital at the University of Münster explicitly focuses on treating early child psychiatric illness, whereas other institutions cover the child protective and legal contexts as well as provide provisions for young children with special needs. However, close cooperation between these types of organizations occurs when necessary.

#### The intermittent treatment concept

Weekly attendance at the Preschool Family Day Hospital in Münster occurs in a two- to three-day blocked structure from Monday to Tuesday or from Wednesday to Friday. Specifically, one patient group attends the Preschool Family Day Hospital with their families on Monday and Tuesday, and they spend the remainder of the week at home. The other patient group attends the hospital from Wednesday to Friday with their families. Consequently, patients and their families experience relatively short sessions of intense therapeutic interventions (two respectively three days), combined with longer sessions of testing changes at home, kindergarten or in playgroups (five respectively four days). This approach has strong ecological validity; at the same time, it is cost-effective because it combines a relatively long treatment period with relatively few but intense treatment days spent in the unit. In our experience, therapeutic change needs time. The regular rhythm of presence and absence from the hospital over approximately two to four months allows the children and their families to mentally process their treatment experiences, to generalize and transfer their treatment results into everyday life, to maintain their social contacts and to integrate into their peer group. The therapists regularly monitor treatment success in external contexts such as kindergarten.

#### Descriptive data

In our research sample, 229 children have been treated in the Preschool Family Day Hospital from 2001 to 2011, comprising 165 boys (72.1%) and 64 girls (27.9%). The average age of the children was 4.56 years (SD_age_ = 1.51; age range = 0.4 -7.8 years). The most common primary diagnoses were *emotional disorders with onset specific to childhood* (50,7%; ICD 10 F93) and *mixed disorders of conduct and emotion* (19,7%; ICD 10 F92). The average number of treatment days was 51,1 (SD_treatment days_ = 23,4; range of treatment days = 5 – 131 days). However, note that the actual duration of treatment is longer than the merely adding up of the treatment days due to the intermittent treatment modus involving attendance of only two respectively three treatment days per week. Therefore, a mean number of 51,1 treatment days corresponds to 22,6 weeks of ongoing treatment (SD _weeks of treatment_ = 13,3) on average. Evaluation data on the clinical outcome of this research sample is processed, but has not yet been published.

### Admission criteria

#### Child admission criteria

All referrals to the Preschool Family Day Hospital are assessed during an initial child psychiatric outpatient appointment to determine the severity of their disorder and whether they need out- or inpatient treatment at a psychiatric day hospital. Contraindications for admission include pervasive developmental disorders such as autism and severe learning disabilities without primary symptoms of psychiatric disorders.

#### Adult admission criteria

Parents are admitted either as accompanying persons or as psychiatric patients in their own right. In Münster, general or private health insurance systems fully pay for their treatment in the Preschool Family Day Clinic as a commendable exception from the general practice where health insurance systems tend to cover the costs of treatment for the child as index patient only, not for attending parents. Parents are not admitted for treatment at the Preschool Family Day Hospital if they suffer from acute psychosis, severe addictive conditions or acute suicidal ideations that require inpatient treatment. In acute and emergency cases, parents may be admitted immediately to the neighboring adult inpatient unit; however, they are able to join their child when they no longer present a danger to themselves or others. If required, both parents and other significant caregivers may take part in all therapeutic interventions on selected days either simultaneously or alternately.

#### Sibling admission criteria

Preschool siblings of the index child may be admitted as patients in their own right or as family members. Their presence constitutes an important contextual factor of the child’s psychiatric treatment and contributes to the ecological validity of the clinical setting [38].

#### Therapeutic team

A senior consultant in child and adolescent psychiatry supervises the Preschool Family Day Hospital. Additional team members include a child psychiatric intern, a developmental psychologist, an occupational therapist, a psychomotor therapist, a child psychotherapist-in-training, three qualified nurses who manage the family during the day and an associated day center nurse with clinical and technical expertise in video therapy assisting in the cutting and editing of relevant video sequences for therapeutic and scientific purposes. Regular case discussions and reviews are held with the department head. The team follows up with all families (i.e., outpatients) after discharge.

### Diagnostic assessment and treatment

#### Child psychiatric assessment

The first two to three day block after admission is usually an intensive diagnostic observation and an assessment of the child’s psychiatric symptoms in the context of parent–child, staff-child and peer-child interactions. Members of the treatment team observe and videotape the child in various contexts. These contexts include structured and unstructured settings; individual behavior and dyadic play episodes with a parent, sibling or member of the treatment team; separations and reunions with attachment figures; family contexts such as shared meal times, playing, and bathroom situations; and peer-group play. Videotaped interactions serve as the central diagnostic/treatment instrument for video-feedback sessions (for an introduction into interactional coaching and the use of video therapy, see [39]). Videotaped patterns of relevant psychiatric symptoms, behaviors and interactions are presented and discussed in the first assessment discussion of the case that involves the entire treatment team. The staff comes to a consensus on a semi-quantified symptom list. After this initial assessment, additional videotaped presentations and reassessments of the case are scheduled at least every 6 weeks, which allows the team to clinical monitor the ongoing treatment. In addition, kindergarten teachers provide written reports or are invited to the Preschool Family Day Hospital for supplemental in-depth information regarding the child’s symptoms. If necessary, children are sent to other University departments, such as General Pediatrics, Pediatric Nephrology, Pediatric Phoniatrics and Pedaudiology or Genetics, for additional medical assessments.

#### Parent psychiatric assessment

All parents who attend the Preschool Family Day Hospital as psychiatric patients undergo an intense exploration of their present psychiatric symptoms and an additional in-depth psychiatric history that includes a medical examination that focuses on the physical and neurological aspects of psychiatric diseases. Additional psychiatric assessments as well as neurological and medical procedures are conducted as required to determine appropriate diagnoses and treatment procedures.

#### Attachment and relationship assessment

As part of the comprehensive interactional family psychiatric assessment, the treatment team rates the quality of the parent–child relationship at entry to and before discharge from the unit. The instruments used in this assessment include the Parent Infant Relationship-Global Assessment Scale (PIR-GAS) [40] and the Relationship Problems Checklist (RPCL) [40]. The former scale quantifies the severity of relationship disorders, which enhances relationship assessment objectivity; the latter scale offers a standardized description of specific types of relationship disorders. When appropriate, attachment patterns are assessed using the Strange Situation Paradigm [41] or, for younger infants, the Still Face Paradigm [42].

### A framework for treatment and therapeutic techniques

Subsequently, a hierarchy of therapeutic goals is developed based on the results of the comprehensive assessment, and appropriate treatment procedures are established in a specific treatment plan. At various stages, each treatment includes a careful reassessment of the child’s psychopathology as well as the contextual parental protective and risk factors that might require changes or adjustments to the treatment plan for both the child and their family.

### Daily treatment structures

**Figure 1:**
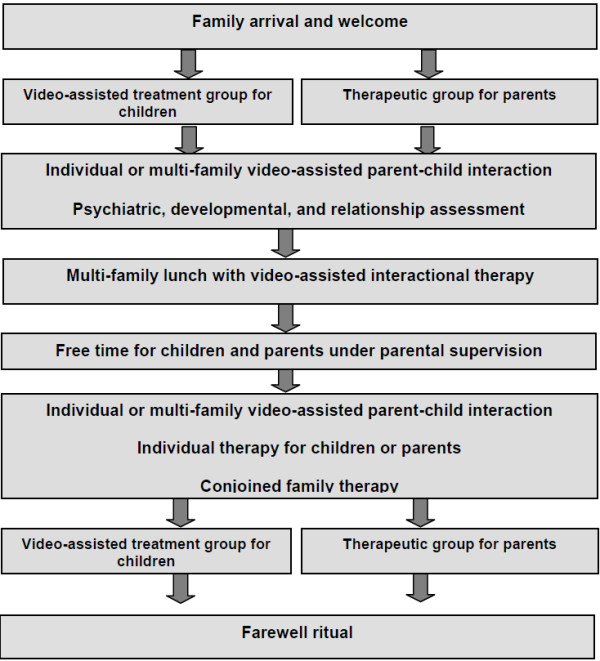
The daily treatment structure of the Preschool Family Day Hospital.

Individual treatment components are implemented at the Preschool Family Day Hospital in the context of a daily routine as illustrated in Figure 1. This routine has recurring procedures that support the establishment of therapeutic rules and a sense of predictability for the child and their family [43], yet its structure is flexible enough to account for the specific assessment and treatment needs of the individual child. The ritualized orientation of a daily structure with clear and predictable transitions from one treatment setting to another can have an important therapeutic effect for the young child [28,44] because predictability and ritualization promote cognitive orientation, reduce anxiety and affect regulation.

After arrival and welcome, each day begins with a separate group treatment session for parents and children. These group sessions are followed by videotaped and video-assisted child–parent interaction treatments that usually occur in the presence of the other families but may be conducted in a separate room as needed. Alternatively, patients may receive one or more of the following: additional psychiatric assessments and treatments; developmental assessments and support; individual treatments for children, parents, or both; and conjoint treatment sessions with both parents. If required, both parents and other significant caregivers may take part in all therapeutic interventions on selected days either simultaneously or alternately. The different treatment components are described below based on the setting, content, task and function of the treatment professionals.

### Child treatment groups

#### Setting

Group treatments for children occur in the morning and the afternoon parallel with a parent treatment group. Accompanying siblings are included. The children patients are separated from their parents for the parallel group sessions but reunited at the end of the sessions. If separation is impossible for the child, they may initially stay with their parent in the parent treatment group. Infants may stay with their parents.

#### Content

The therapeutic children’s group starts in the morning with a common breakfast, and the afternoon session begins with a fruit snack in a shared setting. These meals not only supply basic physical needs after arrival and before departure, but they also offer a social group ritual that is highly relevant for diagnostic and treatment purposes. Following the structured breakfast, free play or psychomotor treatments are offered to all patients. The treatment professionals are advised not to structure free play, which takes place in either the group room or outdoor playground, Treatment professionals are advised to do no more structuring than necessary for assessment or therapeutic purposes which allows the treatment professionals to assess and treat the interactive or emotional deficits of individual patients. Children with developmental disorders may receive individual developmental support during free play. Children with socio-emotional deficits may receive individual social skills training. The specialized psychomotor treatment focuses on physical activities that improve sensory self-awareness, sensory integration, and motor skills.

#### Aims

The separation and reunification of parents and their children are highly relevant for diagnostic and treatment purposes because these situations are stressful for young children and may provoke the open display of psychiatric symptoms, insecure attachments or interactional disorders. Both separation and reunification often allow treatment professionals to disentangle the child and parental factors that contribute to the child’s symptoms.

At the children’s group breakfast, these patients must learn to master emotional regulation by following rules, taking turns and completing simple tasks. These conventions also have strong therapeutic effects for children with disordered feeding and eating behaviors who might benefit from social learning in a group situation. Moreover, the communal meals often help to desensitize children to previously avoided foods. Therapeutic support during free play fosters children’s cognitive orientation and their ability to express evaluative emotions. Furthermore, this support enhances emotional regulation, social cooperation and conflict resolution in an age-appropriate group setting. Psychomotor treatment increases psychomotor coordination and children’s pleasure in healthy physical activity. Exercise enjoyment is often a precondition of cognitive, social and emotional treatment success. The specialized psychomotor treatment addresses children’s fear of physical harm and of other children as well as the overestimation of their own abilities and the underestimation of hazardous situations. The core functions of the therapeutic children’s group are to enhance the children’s social, emotional and self-regulatory skills regarding stress and to foster their self-efficacy to enable healthy self-development. This helps young patients to develop strategies to cope with stress from conflict, disagreements, delayed gratification, anxiety, the experience of rejection, anger, aggression, and so forth. The final aim of this therapy is to assist children in developing age-appropriate strategies to regulate their emotions when alone and in social situations.

#### The function of the treatment professional

The treatment team intervenes during free play only for therapeutic treatments and to assist children in resolving social conflicts. First, they help the children to recognize their own emotions. Second, they assist them in channeling their stress appropriately. Older children are taught to verbalize their emotions. The treatment professionals help patients to develop verbal and non-verbal coping strategies to regulate emotions and social interactions.

### Parent treatment groups

#### Setting

All parents participate in two group sessions per day without their children: one session in the morning and the other in the afternoon. No distinction is made between accompanying parents and adult psychiatric patients because all parents have resources and difficulties to varying degrees. This session aims to assist the child’s treatment via the parents. Thus, this session focuses on the role of parents and not on the self or pathology of the adult. The treatment team moderates and structures each session to ensure that each parent actively participates in the group process.

#### Content

The group setting allows the parents to speak openly regarding their practical difficulties, feelings, experiences, and concerns. Parents are encouraged to help each other and exchange information. The central focus of the morning session is to determine specific treatment goals for each child that day, especially with regard to the video-assisted child–parent interaction treatment units. The afternoon session allows the professionals to evaluate the daily treatment units. The relationship between the behaviors of parents and children is analyzed based on concrete daily episodes.

#### Aims

The exchange among parents with regard to self-help is a core element of this treatment group. Specific aims of the group treatment include emotional support, relief and social learning from other parents’ experiences. Participants learn useful parenting and coping strategies with regard to their psychiatrically ill child from others; furthermore, they adopt realistic treatment expectations about their child’s behavior and development. This adjustment often requires changes in behaviors, expectations and attitudes. An important component of self-help is that parents may accept advice from a peer more readily than from a treatment professional. Parents in this group often benefit considerably from factors such as group cohesiveness or group-derived self-esteem [45]. These factors can enhance the emotional sensitivity and availability of parents, activate their emotional resources and affective coping skills with regard to their child’s psychiatric disturbance, and improve the child’s mental health.

The primary aim of the afternoon evaluation is to develop a deeper understanding of the child’s developmental and mental health problems, particularly in terms of affect regulation, attachment needs, exploratory behaviors, self-efficacy and autonomy. These reflections often assist parents in assuming the role of co-regulators of their child’s arousal, anxiety, and anger as well as other emotions that trigger dysfunctional attachment behaviors in the context of psychiatric symptoms. The transfer of the treatment from the hospital to the home is an important component of intermittent therapy. By focusing on recent episodes of effective sensitivity and emotional availability, parents are encouraged to continue to pursue their goals in intermittent therapy, especially in the final afternoon session at the end of the week.

#### The function of the treatment professional

In the morning session, the team helps the parents to determine realistic treatment goals and strategies for the subsequent sessions of the day. In the afternoon session, the treatment team helps the parents to evaluate whether the therapeutic goals of the day were obtained or whether they might be modified to encourage them to consider issues of transfer for the rest of the treatment block.

### Video-assisted child–parent interactional treatment

#### Setting

Child–parent interaction is a core component of the treatment. This treatment includes an initial and continuous assessment/treatment that occurs twice daily. In general, the treatment occurs in a large multi-family treatment room unless an alternative is required. This room is divided into different areas for specific activities such as psychomotor therapy, role-playing or parlor games. Different situations can trigger or maintain child psychiatric symptoms, including parent–child interactions, peer interactions, individual play, separations and reunions, mealtimes, toilet training, and so on. At the Preschool Family Day Hospital, any of these scenarios might become the focus of an interactional therapy, which accounts for the high ecological validity of this treatment approach. Apart from the large multi-family treatment room, interactional therapy might take place in the day clinic’s kitchen, eating room, bathroom, outdoors, at the day clinic’s playground or in surrounding areas that involve activities such as shopping or traveling downtown. The complex and demanding lunchtime situation with all the families has an especially high clinical and ecological value for the individual and family assessments and treatments. Members of the treatment team closely accompany, structure and support this videotaped setting for assessment and treatment.

#### Content

The direct interactional treatment focuses on the play and daily life activities between children and their parents either individually or in a multi-family group. Parents work toward individually specified treatment goals that were chosen at the morning group treatment to enhance emotional, social or cognitive coping strategies with regard to their children. Videotaped interactional assessments and treatments in the context of a multi-family setting is another core element of the psychiatric treatment modalities of the infants, toddlers and preschoolers at the Preschool Family Day Hospital.

#### Aims

The semi-open treatment setting enhances sharing, openness, and support among the families in the treatment group. This technique constitutes an important aspect of the therapeutic self-help component; moreover, it is often used again for settings such as the parent group, mealtime support, and others. Therapeutic interventions aim to create positive interactions, affect regulation, self-development, mutual emotional availability, and a cognitive-socio-emotional fit between parent and child. Furthermore, these interventions aim to foster communicative, cooperative and conflict-resolution skills to improve the relationship between parent and child. Improved emotional, executive, and communicative skills in parents, children, or both might improve symptomatic behaviors as well as increase affective control in children.

### The task of the treatment professional

Parent’s emotional reactions and behaviors are directly visible, and their attitudes and mental representations are displayed in interactional behavior, which is verbally accessible to the treatment professional. The treatment professional observes parent’s behaviors and interactions in both ordinary and stressful situations, provides feedback and helps them to cope with and behaviorally or cognitively modify stressful parent–child interactions. When the parent–child relationship is severely disturbed by hostility, emotional distance or other forms of insensitive emotional behavior, the treatment professional primarily focuses on identifying less stressful situations that can be enjoyed by children and their parents, such as lying in a hammock together, to initially disburden and eventually enhance the relationship. Parallel treatment sessions with only the parents then take priority to explore and modify emotional attitudes and behavior patterns, originating either from the present family or from the parental family of origin.

The decision making capability of parents who have difficulties with setting boundaries is supported via implemented structures, routines, rituals and rules. The treatment professionals discuss their observations of parenting behavior, attitudes and projections with the family therapist. This therapist subsequently uses this information to modify patient mental representations and belief systems related to lack of parental sensitivity.

### The role of video analysis in interactional treatments

Parent–child interactions in everyday, stressful and contentious situations are videotaped and subsequently analyzed with parents with respect to their cognitive behavioral coping strategies and their emotional attitudes, evaluations and beliefs regarding their children and themselves. The video feedback always begins with successful interactions that emphasize the resources of the parents and children before focusing on dysfunctional patterns of interaction to ease parents into to this therapeutic tool and to strengthen their self-confidence. Various studies have proven the effectiveness of video feedback in the treatment of parents with infants, toddlers and preschoolers [46-48].

### Individual work with children

#### Setting

Individual work with children is an additional, non-routine treatment element. The therapeutic approach of the Preschool Family Day Hospital aims to treat children and their parents together in an integrated interactional multi-family group setting. However, individual work is required for rare, specific cases (e.g., children who have experienced severe violence or extremely destructive patterns of family interactions; those who are severely emotionally burdened, often by a psychiatrically ill parent; or those with disorders such as elective mutism, enuresis or encopresis) that necessitate specific interventions using an individual framework. Usually, these children are removed from the group for individual therapist sessions.

#### Content

Methods of individual therapy include fantasy and role-playing, sand play, play with puppets or other objects, storytelling, and table-oriented play. These methods depend on age, developmental stage, specific symptom requirements, and task- or symptom-oriented interactions with the therapist and are often watched by the parent on video or through a one-way mirror.

#### Aims

Play-oriented therapy allows children to express their feelings and experiences as well as to gain a sense of mastery. Eventually, this therapy helps the child to develop age-appropriate behavioral and emotional skills so that they are able to convey emotions verbally or nonverbally and reach age-appropriate emotional regulation strategies [49]. This technique reduces anxiety and stress [50] and may foster the development of alternative coping skills to reduce stress. Empirical studies support the effectiveness of play-oriented therapy in reducing child psychiatric symptoms [51,52].

#### The function of the treatment professional

The therapist sensitively responds to the child’s play behavior as well as listens to and expands upon the child’s ideas. At times, the child leads this joint activity, whereas at other times, the therapist leads the session. The treatment professional helps the child to express and regulate her or his behavior, underlying stress and emotion to improve emotional balance and develop play skills, a sense of self-efficacy and interactive social skills.

### Individual parent sessions

#### Setting

Individual therapy is offered for all parents once a week in a two- to three-day treatment block, regardless of their status as patients. Children usually do not participate, except in mother-baby therapy.

#### Content

Parental emotional states and biographical experiences have a direct effect on their ability to sensitively understand and regulate their children’s behaviors, intentions and emotions appropriately. If this task is not addressed during the therapeutic process, dysfunctional cognitive, social and emotional processes can be transmitted from parents to their children [53,54]. Therefore, this setting addresses whether parents’ emotional states, their discomfort with the emotional role of being a parent, conflicts with the family of origin, and past traumatic experiences contribute to or exacerbate their children’s symptoms (e.g., “ghosts in the nursery”) as described by Fraiberg, Adelson and Shapiro [55]. If the parents are patients and the psychiatric state requires psychopharmacological interventions, a senior adult psychiatrist prescribes and monitors the effectiveness of medication throughout the treatment.

#### Aims

Individual treatments with parents primarily address their role as parents. The therapeutic dialogue includes the identification and handling of dysfunctional psychological or interpersonal conflicts, developmental counseling, or discussing the possible ways of dealing with their child behaviorally and emotionally in contentious situations as well as situations of parental anxiety, anger, helplessness, overprotectiveness and frustration. This treatment promotes a necessary change in dysfunctional emotional attribution as well as the development of effective parenting behaviors. This change can effectively promote child mental health [56], especially with regard to the appropriate identification of emotional meaning, behavioral coping and socially appropriate adaptations. Parents who are patients receive appropriate psychiatric treatments and learn to distinguish between their conditions and those of their children. Parents learn how their own symptoms affect their child’s mental health.

The central concept in parental therapy is parents’ right to their own unhappiness. Parental experiences with their family of origin might strain them and decrease the likelihood that they will effectively cope with their psychiatrically ill child. Therefore, parents have the right to be unhappy; however, they do not have the right to transmit their unhappiness to their children. The aim of this therapy is to increase parental self-awareness regarding their impulses toward the child. These impulses might be part of their present parent–child relationship or originate from their experience as children with their own parents. This problem can only be solved using a psychotherapeutic treatment that is separate from the child.

#### The function of the therapist

The therapist helps parents to identify inner conflicts, foster self-awareness, and reframe and integrate their relevant life experiences. Furthermore, the therapist helps parents translate these skills into appropriate actions. In addition, the therapist provides developmental, age-appropriate counseling and advice for the parents, including helping to identify child affective states and “read” the child’s practical, cognitive and social needs.

### Conjoint treatment with both parents

#### Setting

Both parents take part in a conjoint session with a family therapist usually every two to three weeks; children usually do not participate.

#### Content

These treatment sessions deal primarily with co-parenting and, if necessary, with partner issues, such as divergent parenting strategies or emotional discord, that interfere with parenting. Primary partner problems are beyond the scope of the treatment and are referred to external marital therapists.

Parenting issues that are relevant to the psychopathology and treatment of the child include conflicts or differences in perceptions, attributions of child behaviors, coping abilities, and emotional regulation strategies. Conjoint sessions focus on fostering a mutual understanding of parental attitudes and actions with the aim of creating or strengthening mutual parental support, especially in situations of need and parent–child conflicts.

#### Aims

These sessions primarily involve the second parent (usually the father) in treatment. This involvement is highly important in the treatment of young children with mental health problems [57]. Both parents are contextual factors who might contribute to the development and maintenance of child psychiatric disorders through inappropriate or dysfunctional parenting behaviors. In addition, parents might contribute to the improvement of the child’s symptoms by changing and adapting their behavior. On the other hand, the child’s symptoms might contribute to the development of dysfunctional parenting behaviors or parental mental health problems. In this regard, parents might develop a shared understanding of the child’s symptoms that enables them to cope with the child’s psychopathology in a shared, consistent and effective manner.

#### The function of the therapist

The therapist identifies and changes the opposing and dysfunctional mental representations of both parents in conflict. These representations might contribute to their child’s psychopathology [58] because they create unpredictability and insecurity as well as evoke feelings of anxiety, confusion, and helplessness in the child. The therapist structures and moderates the session, conveys information and advice, ensures that the concerns of both parents are addressed appropriately, helps parents solve problems and support each other and mediates conflicts when necessary.

## Discussion

This article introduces the Preschool Family Day Hospital for Infants, Toddlers and Preschoolers and their Families at Münster University Hospital, Germany. Specifically, the Preschool Family Day Hospital uses an intermittent treatment approach and an integrated interactional family psychiatric treatment. Family members and relationships may not only be risk factors but also valuable resources that endanger or enhance treatment outcomes, respectively. The treatment therefore includes parents and siblings into the assessment and treatment of the index child when possible. If necessary, parents are also admitted as patients and co-treated in a differentiated but fully integrated family psychiatric approach. German health insurance systems officially recognize and fully fund parental involvement in their children’s psychiatric treatment allowing parents to be included in their children’s therapy.

**Figure 2:**
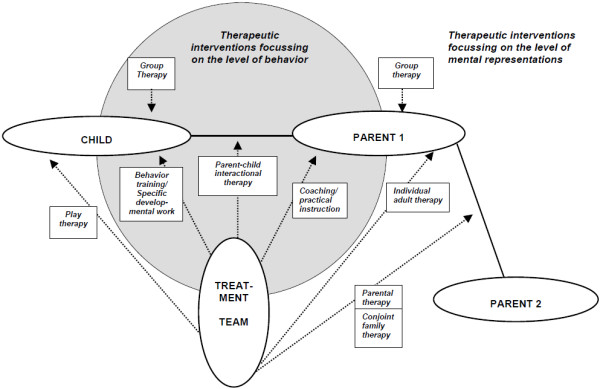
**The multimodal multifamily treatment at the Preschool Family Day Hospital, Münster expands the “ports of entry” [**[59]**,**[60]**] (Stern, 1995; 2004) and Focal Family Theory [**[62]**] (Furniss et al., 1983) approaches.**

The complex assessment and treatment approach used by the Preschool Family Day Hospital can be visualized (Figure 2) by expanding Daniel Stern’s [59,60] concept of “ports of entry”. This theoretical framework describes the possible diagnostic and therapeutic interventions into a family constellation in the context of individual infant, toddler and preschooler psychopathologies [59-61]. The “ports of entry” approach and the “focal family therapy” [62] approach differentiate between child therapeutic interventions on the *level of internal mental representations* such as individual attitudes, fears and beliefs, and those on the *level of behavior in the context of observable interaction*, such as feeding, playing, boundary setting, comforting, and so on. The therapeutic interventions offered by the Preschool Family Day Hospital at the University of Münster Clinic operate on both levels by simultaneously focusing on the child, the parent, the child–parent dyad, the parental dyad, the conjoint family unit, the child group, and the parent group (see Figure 2).

The self-help component of the integrated multi-family treatment constitutes a crucial element in the Preschool Family Day Hospital at the University of Münster, Germany. Another specific feature is the two- to three-day blocked structure of weekly attendance (either on Monday and Tuesday or on Wednesday through Friday). This regular pattern of intensive full-day treatments as well as environmental implementations, generalizations and treatment change assessment allows for a more thorough processing of treatment experiences, which enhances the chances of generalization and a transfer of treatment results to daily life. Research shows that children learn more effectively when input is distributed over longer time periods with treatment-free intervals [63]. The attendance rhythm of the Preschool Family Day Hospital also creates family continuity. This rhythm maintains ordinary social contexts that help infants, toddlers and preschoolers to socially integrate into ordinary family life and with peers. It also permits professionals to monitor the treatment progress in different external social settings, including the home environment. In addition, this treatment is easily managed by parents and does not jeopardize their family or professional lives. Therefore, treatment at the Preschool Family Day Hospital at the University of Münster follows a complex, differentiated, integrated, and innovative approach to infant, toddler and preschooler psychiatry by accounting for the specific needs of this population.

## Conclusions

Various child psychiatric approaches have been developed internationally to treat infants, toddlers and preschoolers in different healthcare systems. The clinical experience gained while developing specific approaches may be important across disciplines, especially as early intervention programmes are more and more favoured in mental healthcare. The Preschool Family Day Hospital for Infants, Toddlers and Preschoolers and their Families at Münster University Hospital, Germany is unique in the German healthcare system with regard to its social-service institution division of labor. As described above, maintaining this early child psychiatric treatment unit is only possible with separate assessment and treatment units for at-risk young children, abused children and their families. These units were initiated and created in parallel. Furthermore, the Preschool Family Day Hospital uses an intermittent treatment approach and an integrated interactional family psychiatric treatment to treat children and their parents as separate patients. This approach includes components of group treatments with children and separate treatments with parents. Specific techniques include video-assisted treatments of the parent–child interaction, psychiatric and psychotherapeutic treatments for parents, and conjoint family therapies that include both parents and siblings. This specialized treatment in a child psychiatric family day hospital is still rare. Family day hospitals with a multidisciplinary, developmentally and family-oriented approach offer themselves as an integrated approach to preschool child psychiatry adapted to the ecological needs of this age group. However, research on this type of treatment for infants, toddlers and preschoolers is sparse [64] underlining the need for further clinical evaluation with quantitative means.

## Competing interests

The authors declare that they have no competing interests.

## Authors’ contributions

TF made substantial contributions to conception and design of the study as well as being the author responsible for the basic treatment design and structure of the preschool family day hospital setting and underlying treatment strategies. In addition he was involved in the implementation and the delivery of the service and in finalising the manuscript. SA and CP drafted the manuscript and contributed to design and conception of the study. JMM and SA performed all statistical analyses. JMM and IW helped with interpretation of data and drafting of the manuscript. MAH and CP played an active role in implementing, service delivery and data gathering. All authors provided a critical revision of the draft for important intellectual content and all authors read and approved the final manuscript.
